# In Vitro Assessment of Gold Nanoparticles on Telomerase Activity and Telomere Length in Human Fibroblasts

**DOI:** 10.3390/ijms241814273

**Published:** 2023-09-19

**Authors:** Xuesheng Han, Alice Hirschel, Menelaos Tsapekos, Diego Perez, David Vollmer

**Affiliations:** 1 Scientific Research Division, 4Life Research, Sandy, UT 84070, USAdavidv@4life.com (D.V.); 2 Life Length SL, 28010 Madrid, Spain

**Keywords:** gold nanoparticles, AuNPs, telomerase activity, telomeres, anti-aging, supplements

## Abstract

Telomerase activity coincides with lengthening of the ends of chromosomes known as telomeres. Telomere length is used as a marker for cellular aging. Telomeres shorten over time as cells divide, and certain bioactive compounds such as gold nanoparticles (AuNPs) may slow the shortening of telomeres by increasing telomerase activity. The objective of the present study is to assess the effect of AuNPs on telomerase activity and telomere length in human fibroblasts. Telomerase activity was measured using enzyme-linked immunosorbent assay (ELISA) in primary human lung fibroblasts (IMR90) and using quantitative PCR-based telomeric repeat amplification protocol (Q-TRAP) in primary human dermal fibroblasts, neonatal (HDFn). Telomere length was determined by Telomere Analysis Technology (TAT^®^)assay in HDFn. In IMR90, all AuNP treatments showed significant increases in telomerase activity when compared to earlier passages. HDFn treated with AuNPs at 0 ppm, 0.05 ppm, 0.5 ppm, or 5 ppm did not show significant differences in telomerase activity compared to the control group. Significant differences in telomere length in HDFn were observed at 2 weeks of 0.05 and 0.5 ppm AuNPs under oxidative culture conditions as compared to the control group. The study showed preliminary evidence that AuNPs may increase telomerase activity and decelerate the shortening of telomeres in human fibroblasts, suggesting its potential anti-aging effects, which warrants further investigation.

## 1. Introduction

Aging is associated with shortened telomeres. Telomeres are protein–deoxyribonucleic acid (DNA) complexes protecting the ends of chromosomes and stabilizing chromosomes. Telomeres do not fully replicate during cell division, causing telomere shortening [1]. Telomerase is an enzyme that, when active, adds DNA sequences to the ends of telomeres to help maintain their length. Telomerase appears to be active in germ cells and some adult stem cells [2,3]. Cells stop dividing and enter senescence once telomeres become critically short [4]. Consequently, telomere length is a valuable marker of cell aging.

Telomere shortening is also correlated with age-related diseases such as cancers, cardiovascular disease, and diabetes [5,6,7,8,9]. Inflammation and oxidative stress have been shown to accelerate telomere shortening in vitro [10,11]. Foods and nutraceuticals such as antioxidants and antioxidant-rich foods, Coenzyme Q10, bovine colostrum, and various essential vitamins and minerals have all been shown to slow telomere shortening, prolong the lifespan of cells, and support healthy cellular aging [12,13,14,15,16,17].

Vitamins and minerals are essential for human health [18]. Ionic and chelated minerals are generally absorbed more efficiently by the human body than elemental minerals [19,20,21]. Recent interest has grown in the additional benefits of ionic elements and minerals. Liquid products containing ionized forms of metals claim to support cellular function since many of these metals are key components in life-sustaining reactions that occur within the human body. Gold nanoparticles (AuNPs) and ionic gold have conflicting evidence showing potential for human benefits, though many of the studies focus on antimicrobial impact [22]. Some studies, however, do focus on the potential antiaging and antioxidant effects of AuNPs [15]. 

The objective of this study was to determine if AuNPs influence cell growth and telomerase activity and slow telomere shortening in two different types of human fibroblasts in vitro. Because oxidative stress is known to accelerate telomere shortening, experiments were conducted under both standard and oxidative conditions in both types of human fibroblasts.

## 2. Results

### 2.1. Toxicity

In IMR90 (primary human lung fibroblasts), a lactate dehydrogenase (LDH) assay was used to examine the toxicity of AuNP treatments. It was determined that AuNPs at concentrations up to 12.5 parts per million (ppm) did not show toxicity, while doses at higher ppm showed signs of toxicity. 

HDFn (primary human dermal fibroblasts, neonatal) viability was determined via the 3-(4,5-Dimethylthiazol-2-yl)-2,5-diphenyltetrazolium bromide (MTT) toxicity assay. At 72-h and 1-week time points, plates were analyzed for cell viability in the presence of all treatment concentrations (up to 5 ppm AuNPs) and growing conditions, and none of the treated cells surpassed 20% mortality during the inoculation periods (Supplementary Information Tables S1–S4). 

Results from the 8-week proliferative analysis assay showed that cells treated with 5 ppm and 0.5 ppm AuNPs had no significant decrease in proliferative capacity compared to the control group under standard conditions and continued from week 3 to week 8. Under oxidative conditions, cells treated with 0.5 ppm AuNPs presented no significant decrease in proliferative capacity compared to the control group from week 3 to week 8. 

All concentrations of AuNPs used subsequently in the study were below the concentrations that showed signs of toxicity, and thus they were non-toxic to IMR90 (up to 12.5 ppm AuNPs) or HDFn (up to 5 ppm AuNPs).

### 2.2. Telomerase Activity

**Figure 1:**
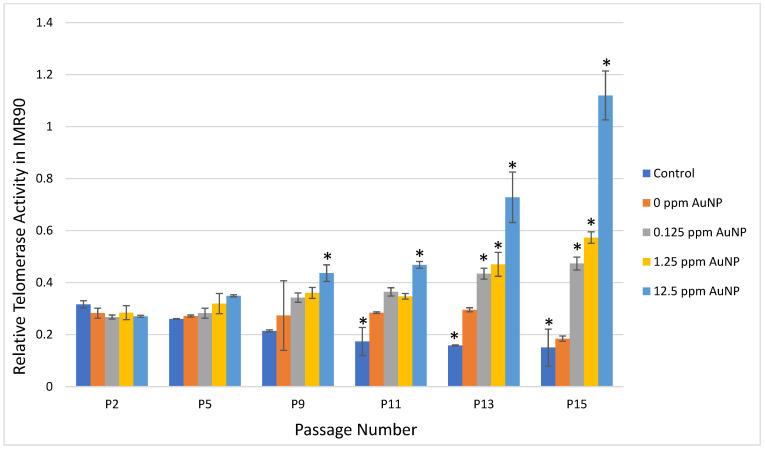
Relative Telomerase Activity in IMR90 Cells at Passage 2, 5, 9, 11, 13, and 15. Data are presented as Mean ± SD and compared to P2, respectively, for statistical significance, which is defined as * *p* < 0.01.

In the Telo TAGGG Telomerase polymerase chain reaction (PCR)–enzyme-linked immunosorbent assay (ELISA), representative passages of 2, 5, 9, 11, 13, and 15 were selected to compare the profile of the telomerase activity. In control cells, telomerase activity was reduced over time as expected because IMR90 cells have a finite lifespan. Comparing Passage 2 (P2) to Passage 11 (P11), Passage 13 (P13), and Passage P15 (P15) in control cells, there was a significant reduction in telomerase activity at each passage (*p* < 0.01) (Figure 1). The 0 ppm AuNP control showed maintenance of telomerase activity for each passage number compared to P2 (Figure 1).

In IMR90 cells treated with 0.125 ppm or 1.25 ppm AuNPs, telomerase activity increased with each passage reaching statistical significance at P13 (*p* < 0.001) and P15 (*p* < 0.0001) when compared to P2 (Figure 1). When treated with 12.5 ppm AuNPs, telomerase activity increased with each passage reaching statistical significance at P9 (*p* < 0.001) and continued through P15 (P11, P13, and P15, *p* < 0.0001) (Figure 1).

**Figure 2:**
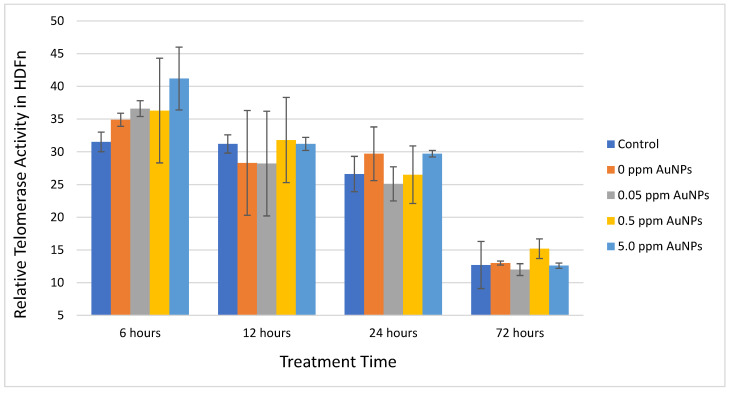
Relative Telomerase Activity in HDFn Cells at 6, 12, 24, and 72 h. Data are presented as Mean ± SD and compared to control treatment, respectively.

In the quantitative PCR-based telomeric repeat amplification protocol (Q-TRAP) assay, HDFn were treated with various concentrations of AuNPs for 6, 12, 24, and 72 h under standard culture conditions. There was no statistically significant difference comparing any treatment condition to the control condition Figure 2). 

### 2.3. Telomere Length

**Figure 3:**
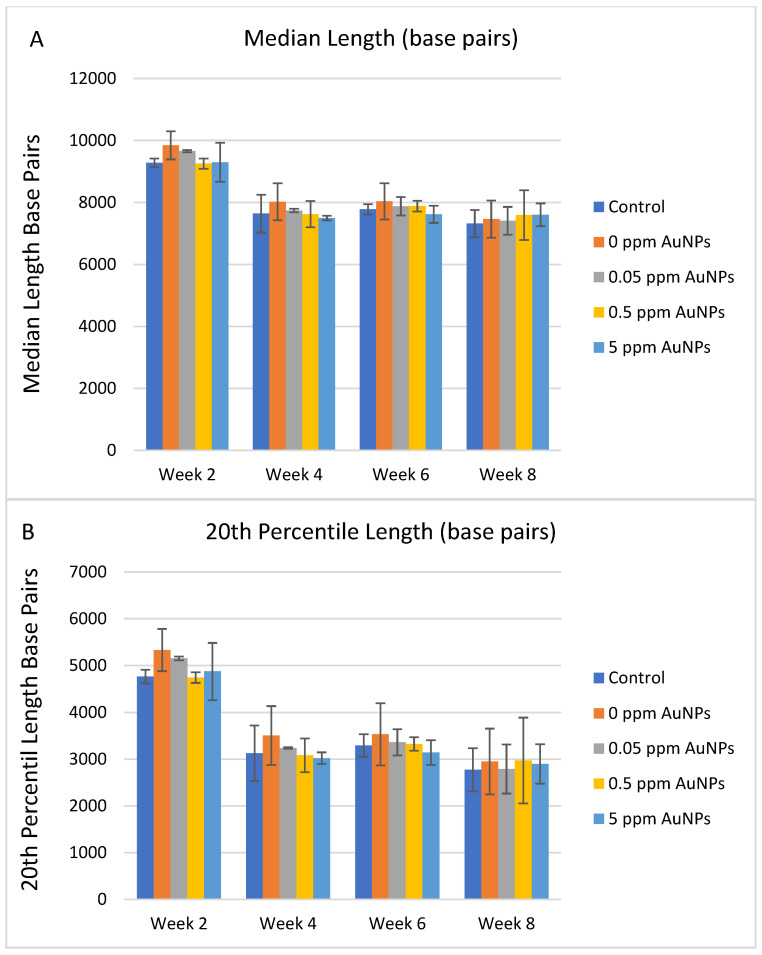

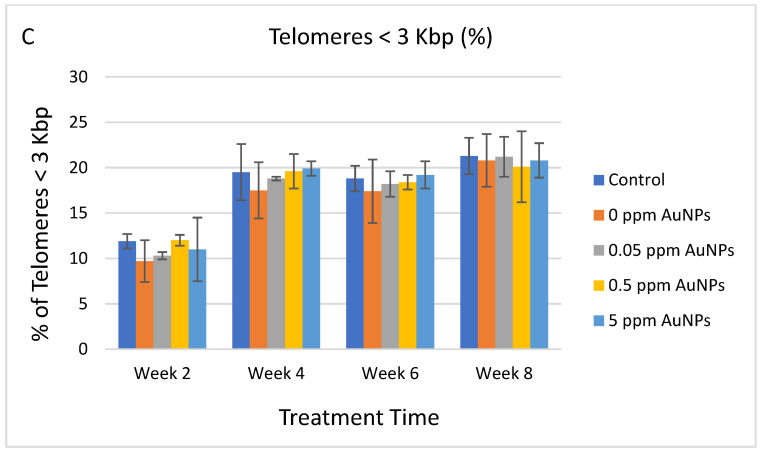
Telomere length in HDFn grown in standard culture conditions. Data are presented as Mean ± SD and compared to control treatment, respectively. (**A**) median length (base pairs); (**B**) the 20th percentile length (base pairs), and (**C**) percentage of telomeres < 3000 base pairs.

**Figure 4:**
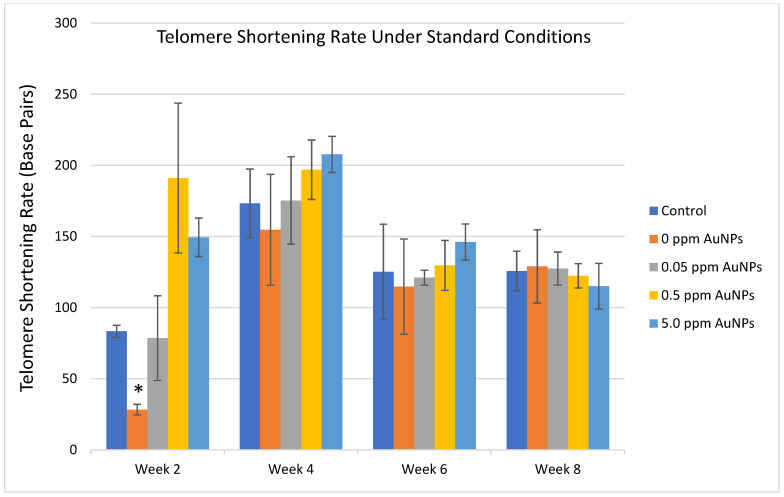
Telomere shortening rate in HDFn under standard non-oxidative culture conditions. * *p* < 0.01 compared to control.

Since cell replication is one of the principal causes of telomere shortening, the telomere length measurements performed were normalized by the population doubling levels (cell replication) in each condition and time point. Under standard culture conditions in HDFn cells, no significant differences in median telomere length, the 20th percentile length, or percentage of telomeres < 3 kilobase pairs (Kbp) were identified between the control group and the treated groups (Figure 3A–C). After normalizing the data by the population doubling, no consistent differences were identified in the telomere shortening rate for any treatment for the entire 8 weeks (Figure 4).

**Figure 5:**
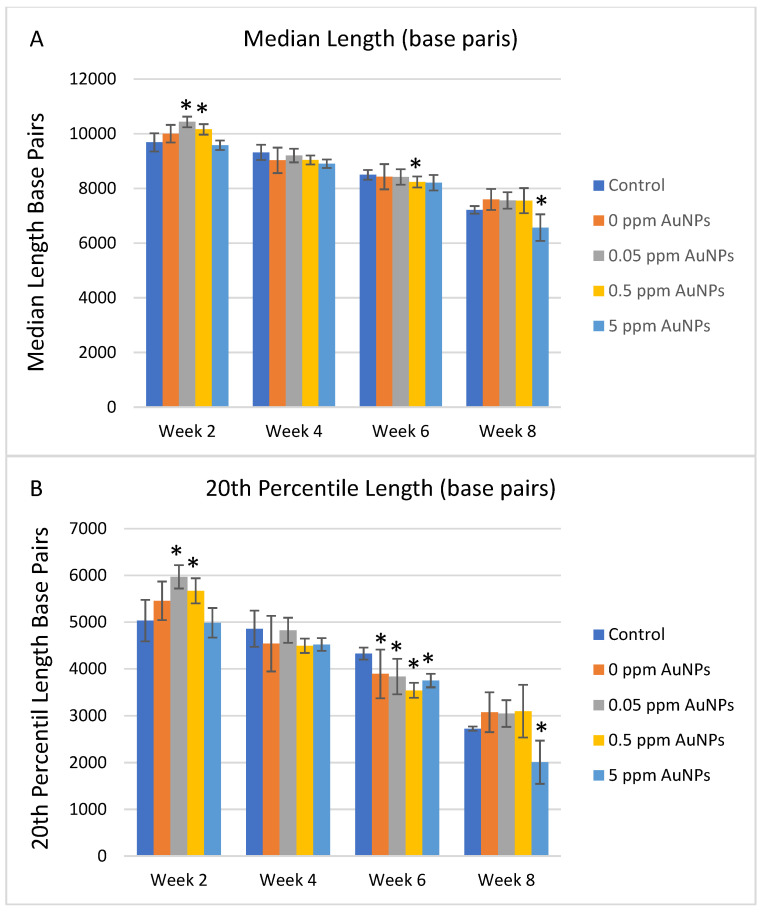

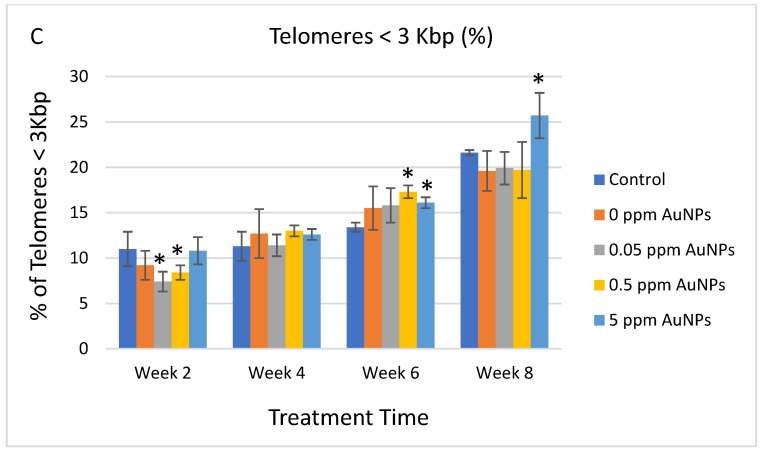
Telomere length in HDFn grown in oxidized culture conditions. Data are presented as Mean ± SD and compared to control treatment, respectively, for statistical significance, which is defined as *p < 0.01. (**A**) median length (base pairs), (**B**) the 20th percentile length (base pairs), and (**C**) percentage of telomeres < 3000 base pairs.

**Figure 6:**
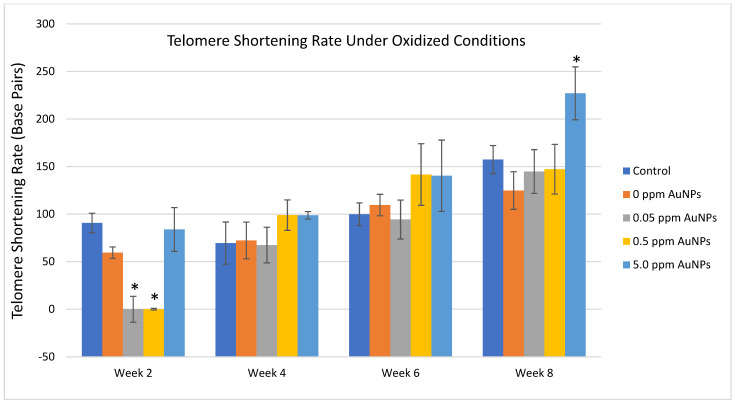
Telomere shortening rate in HDFn under oxidative culture conditions. * *p* < 0.01 compared to control treatment.

Treatment with AuNPs under oxidative cell culture conditions resulted in consistently significant increases in median telomere length and the 20th percentile length and a decrease in the percentage of telomeres < 3 Kbp between the 0.05 ppm and 0.5 ppm AuNPs groups compared to the control group at 2 weeks (Figure 5A–C). Significant decreases in telomere length were observed at various time points and AuNP treatments (Figure 5A–C). After normalizing the data by the population doubling, it is observed that treatment with 0.5 ppm AuNPs reduces the telomere shortening rate consistently throughout the entire expansion. Although these differences were found to be significant only at 2 weeks, a trend can be observed for this concentration. No other consistent differences were identified in telomere shortening rate in oxidative conditions for any treatment for the entire 8 weeks, though 5 ppm AuNPs at week 8 showed a significantly higher telomere shortening rate (Figure 6).

## 3. Discussion

In the present study, AuNP treatment significantly increased telomerase activity in IMR90 cells but did not demonstrate a consistent effect in HDFn cells. Telomere length was also not altered with AuNP treatment in HDFn cells. 

All AuNP treatments tested in the IMR90 cells had significant increases in telomerase activity in later passages of each treatment concentration when compared to its respective P2. Multiple studies have utilized gold nanoparticles as a substrate or component of a technique for measuring telomerase activity in certain models [23,24]. A study analyzing the effects of different nutraceutical formulas followed a similar PCR–ELISA protocol to measure telomerase activity [25]. The results of this study demonstrated that several of these formulas increased telomerase activation, though the increase was greater than that of the AuNP treatment in the present study. Another study utilized zinc, cadmium, and copper to show that metals have the potential to modulate telomerase activity [26]. Recent studies have conversely shown that heavy metals arsenic, cadmium, and lead negatively impact telomere length or may have damaging effects on cells [27]. Telomeric exposure to different metals can have differing effects; an extreme increase in telomerase activity would be considered potentially harmful, whereas a moderate, controlled increase in telomerase activity could potentially benefit cell longevity [28].

Telomerase activity in primary cultures of neonatal human fibroblasts (HDFn) measured by Q-TRAP did not show significant differences as compared to the cells treated with the control condition. The lack of significance at subsequent time points suggests that the provided protective telomeric effect is limited. Q-TRAP is a widely used technique, and recent studies have shown that telomerase activity coincides well with telomeric maintenance in various cellular systems [29]. 

Telomere length measurements by Telomere Analysis Technology (TAT^®^) showed consistent significant differences in HDFn under oxidative conditions after two weeks as compared to the control group for two different AuNP treatment concentrations: 0.5 ppm and 0.05 ppm, while showing no difference under standard, non-oxidative conditions. This is largely consistent with the strong antioxidant activity of AuNPs [15] and suggests that AuNPs might protect telomeres and telomere lengths from oxidative stress during the aging process. This protection might be exerted through the antioxidation pathways in the cells. However, it is also worth noting that higher concentrations of AuNPs under extended oxidative conditions (5 ppm at week 8) seem to increase the telomere shortening rate (Figure 6). These suggest that the effect of AuNPs on telomerase activity and telomere lengths might depend on specific oxidative conditions and stages of cell growth. 

Differences were seen in median telomere length, the 20th percentile length, and percentage of telomeres < 3 Kbp. These are standard methodological markers for Q-FISH analysis of telomere length. A similar study measuring the effects of ergothioneine on telomere length used median telomere length, percentage of telomeres < 3 Kbp, and the 20th percentile length to conclude a role for the amino acid as an antioxidant and support for healthy aging [30]. A meta-analysis specifically compiling data on the role that vitamin D plays in cellular aging discusses conflicting results; though vitamin D appears to regulate the proliferation and senescence, partially through the preservation of telomeres, all published studies have serious limitations, and results do not generally coincide [31]. The results of the multiple assays within the present study acknowledge similar conflicting evidence for the ability of the gold nanoparticle solution to significantly impact telomeric length and function, though the current results merit consideration for the potential to benefit cellular aging.

There is sufficient evidence that AuNPs are safe and nontoxic [32], but the mechanism by which AuNPs could potentially activate telomerase has not been thoroughly investigated. Gold nanoparticles have been shown to catalyze the conversion of nicotinamide adenine dinucleotide (NADH) to NAD+ [33]. A recent study suggests that the increased presence of NAD+ can help stabilize telomeres and improve immune cell function [34]. Supplementation with nicotinamide ribose, a precursor to NAD+, may also help prevent telomere damage since NAD+ is not well regulated when telomeres are short [35]. The concentration of NAD+ also plays a role in the activity of the enzyme tankyrase [36], which assists in the protection of telomeres by increasing telomerase activity [37]. Gold nanoparticles may therefore have an upstream effect on the production of NAD+, which is utilized by these enzymes to provide telomeric support and warrant further investigation. 

## 4. Materials and Methods

### 4.1. Test Articles and Cell Culture

AuNP treatment was given as a liquid supplement: 4Life Elements^®^ Gold Factor™ (4Life Research, Sandy, UT, USA) at varying concentrations. AuNPs are suspended crystalline structures, relatively homogenous with catalytically active surfaces. Each nanocrystal has approximately 68,000 Au atoms per nanocrystal with a 13 nm median diameter (8–28 nm) and a corresponding molar mass of ~1.3 × 10^4^ kDa. AuNPs are suspended in a buffer solution (stabilizer) of 0.04 mg/mL of potassium bicarbonate. More specific information about AuNPs used in the study can be found in this publication [38].

Two different cell types, primary human fibroblasts isolated from the lung (IMR90) and neonatal human fibroblasts (HDFn), were utilized to help elucidate the effects of AuNPs on telomerase activity. IMR90 (ATCC CCL 186) was chosen because these cells have a finite lifespan. Their replicative senescence is induced by critical telomere shortening and limits the proliferation of these cells [39]. Cells were grown as described previously [40]. Briefly, IMR90 was grown for 15 passages. The cells at P1 were passaged into several flasks, allowed to attach overnight, and then treated for 72 h with the AuNP test compound at various concentrations (Table 1). After 72 h treatment, the cells were detached from the cell culture flasks using TrypLE™ Select (Invitrogen, Waltham, MA, USA). Half of the cells were washed and frozen at −80 °C, while the other half were seeded in a fresh flask and treated with the same compound for another 72–96 h. Cells were grown and stored in this manner and treated with each compound for 15 passages. At the end of 15 passages, the cells were collected and processed for analysis.

Primary human neonatal dermal fibroblasts (HDFn, ATCC PCS-201-010™) were also utilized in this study. Cells were seeded at 3 × 10^3^ cells/cm^2^ in a fibroblast medium kit (Innoprot, Bizkaia, Spain). The medium is HEPES and bicarbonate buffer and has a pH of 7.4 when equilibrated in an incubator with an atmosphere of 5% CO_2_/95% air. Treatment compounds under standard culture conditions or oxidative culture conditions (10 µM H_2_O_2_) and vehicle control were added to the cells in culture (Table 2). Oxidative conditions were used as a culture condition as it has been shown to be an effective cell-aged model [10,11]. Media were renewed every 2–3 days, and cells were passaged at sub-confluence (70–80%) every 7 days for 8 weeks. Cell growth was monitored for each condition by counting cell numbers at each passage using a Countess™ cell counter (Invitrogen). Population doubling was calculated as described previously [41]. All treatments and expansions are performed in triplicates to ensure that three physical replicates are analyzed per condition and for each time point.

### 4.2. Toxicity Assays

To examine the toxicity and determine the appropriate treatment dose for each compound in IMR90, the activity of LDH was measured. Lactate dehydrogenase is a stable cytosolic enzyme that is released upon cell lysis. To investigate cell toxicity and death, cells were seeded in 96-well plates and grown overnight, then treated at various doses (Table 1) for 24 h and 72 h. Extracellular LDH released in culture media was measured using an enzymatic reaction as described previously [40]. Briefly, after treatment with the test compound, 50 µL of the cell culture media was transferred to a new 96-well plate, and 50 µL of the reaction mixture was added and incubated at room temperature (RT) for 30 min. Next, 50 µL of stop solution was added, and the plate was read at A450 nm. For all compounds, the subsequent dose used for the study was approximately the highest dose without any evidence of toxicity.

The MTT toxicity test is a colorimetric assay to measure cells’ metabolic activity and serves as a surrogate marker of cell viability in cultured fibroblasts [42,43]. Briefly, HDFn cells previously expanded were seeded in 96-well plates (Nunc) at 0.5 × 10^4^ cells/plate for 72 h of treatment and 0.35 × 10^4^ cells/plate for one week of treatment. Twenty-four hours from seeding, cells were washed once with phosphate-buffered saline (PBS) and treated with the respective compounds (Table 2) in standard culture conditions or oxidative culture conditions. Following treatment, plates were incubated for 72 h and one week, having the medium with treatment changed every two days. After the treatment period, cells were washed twice with PBS, and media were replaced with MTT reagent at 0.5 mg/mL in Dulbecco’s modified eagle medium without phenol red. The plates were gently shaken and incubated for 4 h. After the incubation, the medium was removed and replaced by dimethyl sulfoxide. The plates were gently shaken to solubilize the formazan crystals [44,45]. Absorbance was measured using an Envision multi-plate reader at a wavelength of 570 nm. 

### 4.3. Telomerase Activity

To test the effects of the AuNP treatment compounds on telomerase activity in IMR90, cells were treated with each test compound for 15 passages as described above. The Telo TAGGG Telomerase PCR–ELISA (Roche Applied Science, Penzberg, Germany) was performed on the samples collected at P2, P5, P9, P11, P13, and P15 to investigate the telomerase activity profile over time. Telomerase activity in IMR90 cells was measured in a two-part procedure; the first part is a PCR-based method, and the second part is an ELISA per the PCR–ELISA kit instructions, as described previously [40]. 

To determine the effects of the AuNP treatment compounds on telomerase activity in HDFn, cells were plated as described, and telomerase activity was measured by Q-TRAP [46,47,48]. Telomerase activity was determined in whole cell lysates by Q-TRAP in HDFn as described by Samuel, et al., 2022 [30]. Cells were collected after 6, 12, 24, and 72 h of treatment at the concentrations in Table 2, under standard culture conditions. All samples were run in triplicates. 

### 4.4. Telomere Length

Telomere length measurements were performed using Life Length’s proprietary Telomere Assay Technology (TAT^®^). The TAT^®^ measured telomere length using a high-throughput Q-FISH technique as previously described [49]. The method is based on a quantitative fluorescence in situ hybridization method modified for measuring individual telomeres in cells during interphase. The TAT^®^ measured telomere length in absolute base pair units and provided an assessment of the telomere length distribution through the following three variables per sample: the median telomere length (50th percentile), the 20th percentile telomere length (a second telomere length measurement towards the shorter telomeres), and the percentage of telomeres < 3 Kbp, which is indicative of the quantity of critically short telomeres in the sample. Shorter telomeres are closely associated with cell senescence, which is why the two latter variables are relevant. All samples were run in quintuplicates. HDFn cells were seeded in clear bottom black-walled 384-well plates at the density of 15,000 cells per well of each compound and condition (Table 2). Cells were collected at 2, 4, 6, and 8 weeks for TAT^®^ analysis.

### 4.5. Data Analysis

A 2-way ANOVA was performed for multiple comparisons. Statistically significant differences were defined as a *p*-value of <0.01.

## 5. Conclusions

The present study focused specifically on the change of telomerase activity and various telomere length markers in human fibroblasts by treatment with varying concentrations of AuNP solutions. Results by PCR–ELISA in IMR90 showed significant increases in telomerase activity in AuNP-treated cells over the course of 15 cell passages. In vitro proliferation and telomere length analysis by high-throughput Q-FISH in HDFn showed that a significant potential protective effect was only seen at 2 weeks of treatment with gold nanoparticle solutions at 0.5 and 0.05 ppm AuNPs under oxidative culture conditions in HDFn cells. There is evidence that gold nanoparticle solutions may increase telomerase activity in vitro; however, the direct impact the telomerase activation has on actual telomere length measurements may require additional research.

## Figures and Tables

**Table 1:** Treatment compounds and doses utilized in IMR90 experiments.

Treatment Dose	Compound
0 ppm	Control
0 ppm	Control, with AuNP buffer
0.125 ppm	AuNPs
1.25 ppm	AuNPs
12.5 ppm	AuNPs

**Table 2:** Treatment compounds and doses utilized in HDFn culture experiments. Cells were treated under standard culture conditions and oxidative (10 µM H_2_O_2_) culture conditions.

Treatment Dose	Compound
0 ppm	Control, no treatment
0 ppm	Control, with AuNP buffer
0.05 ppm	AuNP
0.5 ppm	AuNP
5.0 ppm	AuNP

## Supplementary Materials

The following supporting information can be downloaded at: https://www.mdpi.com/article/10.3390/ijms241814273/s1.
