# Supplementary material for: In Vitro Assessment of Gold Nanoparticles on Telomerase Activity and Telomere Length in Human Fibroblasts
Source: Int J Mol Sci. 2023 Sep 19;24(18):14273. doi: 10.3390/ijms241814273 (PMC10532081; doi:10.3390/ijms241814273)
Supplement: Supplementary file 1 [file ijms-24-14273-s001.zip › ijms-2539232-supplementary.pdf]

Supplementary Information

Toxicity Assay (MTT) of Gold Nanoparticles (AuNPs) in primary fibroblast cells (HDFn) at 72 hours and one week.

Triplicates per concentration were tested under standard as well as oxidative conditions to evaluate the effect of AuNPs at different concentrations. MTT results showing cell death values above 20% for all measurements are considered as having a significant toxic effect. MTT results showing cell death values below 20% are considered non-toxic.

Table S1. MTT assay results expressed as the percentage of cell death after 72-hour treatments under standard condition.

72 hours - Standard condition					
**Concentration (μg/ml)**	**Cell death #1 (%)**	**Cell death #2 (%)**	**Cell death #3 (%)**	**MEAN**	**SD**
**5 ppm**	-19.0	-34.3	-11.7	-21.7	11.5
**0.5 ppm**	3.1	1.2	5.8	3.4	2.3
**0.05 ppm**	-5.1	4.7	11.8	3.8	8.5
**0 ppm**	11.7	12.0	5.9	9.8	3.5

Table S2. MTT assay results expressed as the percentage of cell death after 72-hour treatments under oxidative condition.

72 hours - Oxidative condition					
**Concentration (μg/ml)**	**Cell death #1 (%)**	**Cell death #2 (%)**	**Cell death #3 (%)**	**MEAN**	**SD**
**5 ppm**	-4.5	5.2	1.8	0.8	4.9
**0.5 ppm**	12.8	9.2	26.5	16.1	9.1
**0.05 ppm**	6.7	13.8	-4.7	5.3	9.4
**0 ppm**	-2.4	-0.2	9.0	2.1	6.1

Table S3. MTT assay results expressed as the percentage of cell death after 1-week treatments under standard condition.

1 week - Standard condition					
**Concentration (μg/ml)**	**Cell death #1 (%)**	**Cell death #2 (%)**	**Cell death #3 (%)**	**MEAN**	**SD**
**5 ppm**	-27.1	-15.6	-12.5	-18.4	7.7
**0.5 ppm**	9.0	8.4	12.3	9.9	2.1
**0.05 ppm**	12.0	9.0	3.3	8.1	4.4
**0 ppm**	2.4	0.2	6.2	2.9	3.0

Table S4. MTT assay results expressed as the percentage of cell death after 1-week treatments under oxidative condition.

1 week - Oxidative condition					
**Concentration (μg/ml)**	**Cell death #1 (%)**	**Cell death #2 (%)**	**Cell death #3 (%)**	**MEAN**	**SD**
**5 ppm**	-16.2	-4.5	-5.0	-8.6	6.6
**0.5 ppm**	13.6	10.6	14.4	12.9	2.0

**0.05 ppm**	4.4	6.4	3.5	4.8	1.5
**0 ppm**	2.5	2.2	2.2	2.3	0.2
